# Case Report: Severe Hyponatremia in Infants With Urinary Tract Infection

**DOI:** 10.3389/fped.2021.655010

**Published:** 2021-05-05

**Authors:** Karmila Abu Bakar, Muhammad Y. Jalaludin, Nur Zainal, Sze L. Woon, Nurwahida Mohd Zikre, Nurshadia Samingan, Syaza Ab Rahman, Caroline S. Y. Eng

**Affiliations:** ^1^Paediatric Unit, Faculty of Medicine, University Malaya, Kuala Lumpur, Malaysia; ^2^Paediatric Unit, Hospital Tuanku Ja'afar, Seremban, Malaysia

**Keywords:** hyponatremia, hyperkalemia, urinary tract infection, pseudohypoaldosteronism, congenital anomaly of kidney and urinary tract (CAKUT)

## Abstract

**Introduction:** Many reports on investigations and treatments in UTI, however little, have been mentioned with regard to electrolyte abnormalities. Secondary pseudohypoaldosteronism (PHA) in UTI, though less common, is a known association. Features include hyponatremia and concomitant hyperkalemia.

**Objectives:** We aim to highlight these uncommon sequelae in UTI to avoid incorrect diagnosis and unnecessary investigations.

**Study Design:** Clinical data of patients admitted and referred to a pediatric nephrologist at the University Malaya Medical Center between May 2019 and October 2020 were collated and elaborated.

**Results and Discussion:** We report three infants with hyponatremia and hyperkalemia during UTI episodes. Two infants were known to have posterior urethral valve (PUV) before the onset of UTI and one infant had UTI, which led to investigations confirming the diagnosis of bladder vaginal fistula. The electrolyte derangements were temporary and resolved within 48 to 72 h of treatment with intravenous fluid and appropriate antibiotic therapy. Out of three, only one had a hormonal study, which confirms PHA. Reduced aldosterone activity could be due to absolute reduction in aldosterone titer or lack of aldosterone responsiveness at tubular (other tissues) level. In the latter, aldosterone titer is elevated. The infant in our cohort who had hormonal evaluation had the mentioned electrolyte abnormalities with a markedly elevated aldosterone titer. This demonstrated defective action of the hormone at the level of mineralocorticoid receptor. Although the remaining two infants had no confirmatory hormonal study, all of them recovered within 48 h of hospital admission, after receiving appropriate management for the primary problem, which was UTI. We observed a slower recovery of hyponatremia in relation to hyperkalemia, but none of these infants required salt replacement upon discharge.

**Conclusion:** Infants with severe UTI and deranged electrolytes should be screened for structural abnormality and vice versa. Not all infants require hormonal screening, but those who required prolonged salt replacement or showed involvement of other systems warrant further evaluation.

## Introduction

Urinary tract infection is a common childhood disorder. By 7 years of age, 8% of girls and 2% of boys will have at least one episode of UTI (1). In a study of infants presenting to pediatric emergency departments, the prevalence of febrile UTI in infants younger than 60 days was 9% (2).

Electrolyte abnormalities are commonly observed in children with UTI especially among those who are ill and warrant hospitalization. These abnormalities may be contributed by various reasons, commonly following non-specific symptoms such as vomiting, diarrhea, or reduced oral intake. The utility of hypotonic solutions for maintenance intravenous fluid administration in children exacerbates hyponatremia too; however, this practice has changed in many centers worldwide (3). In severe cases, hyponatremia has been cited to reflect the degree of inflammation. Low sodium has been shown to be independently associated with the degree of inflammation in children with febrile UTI. During severe inflammatory processes, pro-inflammatory cytokines such as interleukin (IL1b) reduces expression of apical epithelial sodium channel and sodium ATPase at the basolateral (4). There is also excess of antidiuretic hormone activity with the rise in IL1b (5). These changes during the inflammatory process lead to a decline in serum sodium. This is usually observed as isolated hyponatremia in the absence of potassium abnormality.

Over the years, guidelines on UTI target optimal antimicrobial management, imaging the urinary tracts, and prevention of recurrence in high-risk groups. Little has been mentioned with regard to electrolyte abnormalities in UTI, which may contribute to patient's morbidity and add to diagnostic challenges. Herein, we report a series of three infants with congenital abnormality of the kidney and urinary tract (CAKUT) presenting with severe hyponatremia and concomitant febrile UTI.

## Patients and Methods

Case notes were reviewed critically. All the patients were admitted and referred to a pediatric nephrologist at the University Malaya Medical Center between May 2019 and October 2020. The clinical data of the involved patients were collated and elaborated. We obtained written informed consent from all parents or guardians of the patients involved in the study.

## Case Description

### Case 1

A 2 month-old boy, with an underlying posterior urethral valve presented with an acute febrile illness. His feedings had been normal. On clinical examination, his growth was at the third centile. His anterior fontanelle was normotensive, and the mucous membrane was moist. The cardiorespiratory and abdominal examinations were unremarkable. His urinalysis showed packed field leukocytes, bacteria, and positive leukocyte esterase. He had severe hyponatremia; serum sodium was 119 mmol/L with hyperkalemia, potassium was 6.4 mmol/L, and presence metabolic acidosis. Blood urea and creatinine were 7 mmol/L and 43 μmol/L, respectively. *Extended spectrum beta-lactamase (ESBL) Klebsiella pneumoniae* was isolated from his urine. His blood pressure was 112/60 mmHg. Clinical examination otherwise was unremarkable. He did not show signs of virilization. During the episode of illness, hormonal evaluation showed hyperaldosteronism with normal 17-hydroxyprogesterone [aldosterone of 15,428 pmol/L (102–1,197 pmol/L) and 17-OHP of 3.8 nmol/L (0.97–10 nmol/L)]. The electrolyte abnormalities improved with standard therapy for UTI (administration of intravenous fluid and antibiotic) within 48 h of hospital admission. At 3 months post-infection, his dimercaptosuccinic acid (DMSA) scan showed scarring at the right upper pole with right to left differential function of 31 and 69%.

### Case 2

A 2-month-old girl presented with the first episode of UTI. She had high-grade fever for 3 days associated with foul-smelling urine. She had no vomiting, and her oral intake was normal. She was born full term with birth weight of 3.2 kg. Both antenatal and immediate postnatal periods were uneventful. Her growth had been along the 50th centile. On arrival at the hospital, her BP was 116/66 mmHg. Her anterior fontanelle was normotensive, and she had moist mucous membrane. Apart from a soft systolic murmur, no other abnormalities were detected. Her electrolytes were severely deranged: serum sodium of 116 mmol/L, potassium of 7 mmol/L, and presence of metabolic acidosis. Urine culture grew *Pseudomonas aeruginosa*. Intravenous fluid and antibiotics were instituted. Hyperkalemia and acidosis were resolved within 48 h of therapy, but she needed oral sodium replacement for a further 24 h. Evaluation post-UTI showed right hydronephrosis with an anteroposterior renal pelvic diameter of 1.7 cm and an abnormal connection between the bladder and vagina on micturition cystourethrography (MCUG). She was then referred to the surgical team for corrective surgery. While waiting for the surgery, she suffered another two episodes of UTI despite started on antibiotic prophylaxis. In both episodes, the electrolytes were deranged in the same fashion. After the correction of the vesico-vaginal fistula, no recurrence of UTI was observed, and her electrolytes remained normal. She was planned for a DMSA to assess the degree of her kidney scarring.

### Case 3

An 8-month-old boy was born full term at 38 weeks with birth weight of 3.26 kg. Antenatally, he was diagnosed to have bilateral gross hydronephrosis at 30 weeks of gestation. Postnatal kidney ultrasound showed bilateral gross hydronephrosis with thinning of the cortex. His MCUG suggested a posterior urethral valve, and he had valve fulguration at 6 weeks old. He was thriving steadily within the range of 15th to 50th centile. Otherwise, physical examination was unremarkable. Unfortunately, he continued to suffer from recurrent episodes of UTIs. Typically, he would only present with fever. There was no vomiting or changes in his oral intake. His urine cultures consistently yielded ESBL *Enterobacter cloacae*. In three out of the five episodes, serum electrolytes showed hyponatremia (the lowest was 112 mmol/L) and hyperkalemia (highest serum potassium level at 6.4 mmol/L). These electrolyte abnormalities improved markedly each time with prompt antibiotic therapy and short-term oral sodium replacement. At the time of writing, DMSA was still pending.

## Discussion

Acute hyponatremia is potentially detrimental to health. In an already ill child with febrile UTI, this could compound to disease-related morbidity. In our series, all infants had severe hyponatremia and hyperkalemia at the time of presentation (Table 1). Such “blueprints” reflected a state of reduced aldosterone activity. It was important to ensure that masquerading underneath these biochemical abnormalities was not a potentially life-threatening differential diagnosis of congenital adrenal hyperplasia (CAH). A milder form of CAH that presents much later may not show virilizing signs initially, while those who developed electrolyte abnormalities early should demonstrate some evidence of virilization (6). It is worth noting that all three of our patients presented early within their first year of life, and none showed virilization. This made the possibility of CAH less likely. A normal level of 17 hydroxyprogesterone excludes the possibility of CAH as well.

**Table 1:** Clinical characteristics and laboratory data of infants with severe hyponatremia.

	**Case 1**	**Case 2**	**Case 3**
Age (months)	3	2	8
Gender	Male	Female	Male
Serum Na (mmol/L)	119.0	116.0	112.0
Serum K (mmol/L)	6.4	7.0	6.4
Serum creatinine (μmol/L)	43.0	40.0	40.0
Serum pH	7.18	7.31	7.30
Serum bicarbonate (mmol/L)	14	16	17
Serum chloride (mmol/L)	91	89	85
Serum anion gap	20	19	20
Serum aldosterone (pmol/L)	15,428.0	–	–
Serum 17-OHP(nmol/L)	3.8	–	–
Identified structural abnormality	Posterior urethral valve	Vesico-vaginal fistula	Posterior urethral valve
Organism cultured from urine	*ESBL Klebsiella pneumonia*	*Pseudomonas aeruginosa*	*ESBL Enterobacter cloacae*
Method of urine collection	In–out catheterization	In–out catheterization	In–out catheterization
Organism count (CFU/ml)	>10^5^	>10^5^	>10^5^

Various factors contribute to the development of hyponatremia in UTI. Poor fluid and caloric intake, frequent regurgitations, loose stools, and renal unresponsiveness to aldosterone are among the likely cause (7). In depletional hyponatremia, concurrent hyperkalemia is not observed, or rather, potassium may be low due to the rapid rise of potassium excretion in response to the loss of sodium (8). A complete history taking and thorough physical examination should not be underestimated; although simple, it is important in differentiating possible causes of hyponatremia (9) and to guide on the subsequent management.

Serum sodium in our cohort was measured using indirect potentiometry. Compared with direct potentiometry, the samples are diluted first before in contact with ion-selective electrodes (ISE). Electrolyte concentration in the diluted sample is calculated assuming that plasma water concentration is 93%. We acknowledge that variation in serum sodium exists when determined *via* different laboratory techniques. Milani et al. compared serum sodium analyzed by direct and indirect potentiometry and found that the former is superior. The determinants of incongruity were age, hemoglobin, age and albumin, and albumin and hemoglobin (10). This potential incongruence due to laboratory techniques is minimized by utilizing the same method for serial monitoring.

Low serum sodium and high serum potassium were hallmarks of reduced aldosterone activity. This could be due to absolute reduction in aldosterone titer or lack of aldosterone responsiveness at tubular (other tissues) level (11). In the latter, aldosterone titer is elevated. One infant in our cohort had hormonal evaluation, which supported pseudohypoaldosteronism. He had the mentioned electrolyte abnormalities and a markedly elevated aldosterone titer. This demonstrated a defective action of the hormone at the level of mineralocorticoid receptor (12). Although, the remaining two infants had no confirmatory hormonal study, all of them recovered within 48 h of hospital admission, after receiving appropriate management for the primary problem, which was UTI. We observed slower recovery of hyponatremia in relation to hyperkalemia, but none of these infants required salt replacement upon discharge.

The actions of aldosterone have been well described. At the cortical collecting duct, aldosterone increases the activity of basolateral Na/K ATPase, luminal expression of epithelial sodium channel (ENaC), and the activity of luminal renal outer medullary potassium (ROMK) channels (13). This favors absorption of sodium and excretion of potassium under normal circumstances. Mutations of genes regulating these channels occur in primary pseudohypoaldosteronism (PHA), both type I and type II. Mutations in the epithelial sodium channel (EnaC) lead to autosomal recessive form of PHA type 1, whereas the autosomal dominant form is characterized by mutations in the mineralocorticoid receptor. In PHA type 1, the key clinical features would be hyponatremia, hypovolemia, hyperkalemia, and metabolic acidosis. PHA type II or Gordon syndrome is a resultant of mutations in a family of serine–threonine kinases [with-no-lysine kinases (WNK)1 and WNK4]. WNK4 inhibits expression of sodium chloride (NaCl) cotransporter (NCC). Hence, in PHA type II, mutation of WNK4 increases sodium reabsorption while minimizing potassium loss leading to the syndrome of hypertension and hyperkalemia (13). Secondary pseudohypoaldosteronism has been reported in young infants presenting with UTI with or without urinary tract anomalies, with biochemical abnormalities mimicking that of PHA type 1 (14–17).

Delforge et al. examined a series of 116 infants with secondary pseudohypoaldosteronism and CAKUT. Only 10% showed such biochemical abnormalities in the absence of UTI (11). All the patients in our cohort had underlying CAKUT. Interestingly, the secondary pseudohypoaldosteronism recurred during every episode of UTI, and in the second case, this ceased after urological correction. Our observation concurred with the case series reported in the past. This suggests the importance of screening electrolytes for infants with CAKUT. Vice versa, in young infants with known CAKUT presenting with UTI, electrolyte evaluation is necessary, and it is worth to consider secondary pseudohypoaldosteronism. As the latter is transient, early recognition would allow appropriate management, while we expectantly monitor for recovery with time. In a resource-limited environment like in our centers, this may safely avoid unnecessary endocrinology workup at the first instance. Furthermore, long turn-around time with hormonal testing makes it less feasible to guide on immediate management. Nevertheless, we reckon that infants who showed delayed resolution may benefit from the further hormonal investigation.

In conclusion, the presence of CAKUT in all the cases illustrated in this series supports monitoring of electrolytes in a child with UTI when CAKUT is present.

## Data Availability Statement

The original contributions presented in the study are included in the article/supplementary material, further inquiries can be directed to the corresponding authors.

## Ethics Statement

Written informed consent was obtained from the individual(s), and minor(s)' legal guardian/next of kin, for the publication of any potentially identifiable images or data included in this article.

## Author Contributions

All authors listed have made a substantial, direct and intellectual contribution to the work, and approved it for publication.

## Conflict of Interest

The authors declare that the research was conducted in the absence of any commercial or financial relationships that could be construed as a potential conflict of interest.
